# Transition from Ambrisentan to Bosentan in Pulmonary Arterial Hypertension: A Single-Center Prospective Study

**DOI:** 10.1155/2018/9836820

**Published:** 2018-04-11

**Authors:** Su-Gang Gong, Lan Wang, Bigyan Pudasaini, Ping Yuan, Rong Jiang, Qin-Hua Zhao, Jing He, Rui Zhang, Wen-hui Wu, Jin-Ming Liu, Cai-cun Zhou

**Affiliations:** ^1^Department of Cardio-Pulmonary Circulation, The Third Affiliated Hospital of Soochow University, Shanghai Pulmonary Hospital, Suzhou, Jiangsu 215000, China; ^2^Department of Cardio-Pulmonary Circulation, Shanghai Pulmonary Hospital, Tongji University School of Medicine, Shanghai 200433, China; ^3^Department of Oncology, Shanghai Pulmonary Hospital, Tongji University School of Medicine, Shanghai 200433, China

## Abstract

*Background and objective*: Two endothelin receptor antagonists (ETRAs), bosentan and ambrisentan, are approved for patients with pulmonary arterial hypertension (PAH). However, there is little information about the transition strategy between these two ETRAs. We aimed to evaluate the safety and efficacy from ambrisentan to bosentan. *Methods*: Twenty PAH patients were enrolled into the single-center, open-labelled prospective study. Echocardiogram, WHO functional class (WHO-FC), 6-minute walking distance (6MWD), right heart catheterization, and hemotology were collected. After receiving oral 5 mg ambrisentan daily initially for one year, the patients were divided into two arms: eight patients switched to bosentan, while the remaining 12 patients continued ambrisentan. Characteristics at baseline, 1-and 2-year follow-up points were compared. Results: There were no significant differences in echocardiogram, WHO-FC, hemodynamics, demographics and liver function at baseline, 1-and 2-year points in both arms. 6MWD in bosentan group was significantly shorter at baseline. But there were no significant differences of 6MWD at 1- and 2-year points. Conclusions: It is safe for stable PAH patients to transition from ambrisentan to bosentan without hemodynamic or hematologic deterioration.

## 1. Introduction

Pulmonary arterial hypertension (PAH) is a progressive disease characterized by increasing pulmonary arterial pressure and pulmonary vascular resistance, leading to right heart failure and ultimately death [1, 2]. Endothelin 1 (ET-1), a modulator of pulmonary vascular remodeling, is overexpressed within the remodeled lung vasculatures and contributes to vascular narrowing [3, 4]. ET-1 binds to 2 receptors, ETA and ETB. Endothelin receptor antagonists (ETRAs) that block either A or A and B receptors are used to treat PAH. At present, macitentan is not on the list in China, and sitaxsentan was withdrawn from the market, so bosentan and ambrisentan are the only ETRAs currently available. Both bosentan and ambrisentan have been proved to be safe alternatives to sitaxsentan [5, 6]. But there is little information about the safety of the transition between these two oral drugs in PAH patients. We reported our single-center experience of transiting PAH patients from ambrisentan to bosentan.

Side effects such as liver toxicity and drug interactions differ between these ETRAs [2]. We present available data from 20 stable patients who were on 5 mg daily oral ambrisentan; after 1 year, 8 patients agreed to switch to bosentan, while the remaining 12 patients continued with ambrisentan.

## 2. Methods

### 2.1. Subjects

We performed a prospective single-center study at our center. All patients included are ethnic Chinese (Table 1). Twenty stable PAH patients on 5 mg daily oral ambrisentan were enrolled into the study. Nine patients were treated with sildenafil and continued with it throughout the study. After 1 year, eight patients agreed to subsequently switch to bosentan (started with 62.5 mg bid and increased to 125 mg bid after four weeks if there was no liver toxicity). The remaining 12 patients continued with ambrisentan treatment. The study sustained for two years, and the ongoing therapy was maintained for all the patients after the study. After the switch, patients were grouped as bosentan (*n* = 8) or ambrisentan (*n* = 12) group. Data were collected from each patient at baseline, 1-year, and 2-year points. Collected data included 6-minute walk distance (6MWD), NT-pro-brain natriuretic peptide (NT-proBNP), WHO functional class (WHO FC), echocardiography parameters (tricuspid annular plane systolic excursion (TAPSE), pulmonary artery systolic pressure, LV eccentricity index, pericardial effusion), arterial oxygen saturation, alanine aminotransferase (ALT), aspartate aminotransferase (AST), and uric acid plasma concentrations (UA).

Ethical approval was received from the ethics committee of Shanghai Pulmonary Hospital. All patients consented to be included in the study.

### 2.2. Statistical Analyses

Data are presented as mean ± standard deviation, unless stated differently. Comparisons of characteristics, 6MWD, echocardiography parameters, and hematology between two groups were performed using independent *t* tests for normally distributed data and Mann–Whitney *U* tests for not normally distributed data. *Chi*-square tests were used to analyze changes of pericardial effusion and WHO FC. Statistical analyses were conducted using SPSS 20.0. A *P* value < 0.05 was considered statistically significant.

## 3. Results

Demographic and baseline characteristics are outlined in Table 1. There were 6 females and 2 males with a mean age of 51 ± 12 (mean ± SD) in the transition group, and 10 females and 2 males with a mean age of 39 ± 17 (mean ± SD) in the untransition group. Hemodynamic data for all patients are illustrated in 2. Serum ALT was 24.9 ± 9.2 mU/mL and 29.8 ± 14.4 mU/mL at baseline, 23.3 ± 7.5 mU/mL and 26.1 ± 8.0 mU/mL at 1 year, and 26.6 ± 9.9 mU/mL and 23.0 ± 9.1 mU/mL at 2 year in bosentan and ambrisentan groups, respectively. Serum AST was 26.3 ± 7.2 mU/mL and 21.9 ± 4.9 mU/mL at baseline, 26.3 ± 9.7 mU/mL and 23.3 ± 7.3 mU/mL at 1 year, and 26.9 ± 8.4 mU/mL and 23.5 ± 6.1 mU/mL at 2 year in bosentan and ambrisentan groups, respectively (Table 3). None of the patients discontinued ETRAs due to liver function abnormalities.

### 3.1. Effect of Transition on 6MWD

6MWD was 432.5 ± 79.1 m in the bosentan group and 511.7 ± 41.4 m in the ambrisentan group at baseline, *P* < 0.05. 6MWD was 422.3 ± 81.1 m and 409.0 ± 92.0 m at 1 year and 2 year in the bosentan group, and 517.3 ± 60.8 m and 520.0 ± 72.4 m in the ambrisentan group. 6MWD deviation from baseline to 1 year was 10.3 ± 14.5 m and −5.6 ± 38.9 m, and the deviation from 1 year to 2 year was 13.3 ± 29.6 m and −2.8 ± 21.2 m, respectively, in bosentan and ambrisentan groups (Table 4).

### 3.2. Effect of Transition on Serum NT-proBNP, Uric Acid, D-Dimer, and PaCO_2_

No differences were observed in serum NT-proBNP levels, uric acid, D-dimer, and PaCO_2_ (*P* > 0.05) at baseline, 1 year, and 2 year in two groups (Table 4).

No significant changes were found in deviations from baseline to 1 year or from 1 year to 2 year between bosentan and ambrisentan groups in NT-proBNP, uric acid, D-dimer, and PaCO_2_ (*P* > 0.05) (Table 4).

### 3.3. Effect of Transition on Echocardiography Parameters and WHO FC

No differences were observed in SPAP levels at baseline, 1 year, and 2 year between two groups or in TAPSE and LV eccentricity index (*P* > 0.05) (Table 4). No significant changes were found in SPAP, TAPSE, and LV eccentricity index (*P* > 0.05) from baseline to 1 year or from 1 year to 2 year between bosentan and ambrisentan groups (*P* > 0.05) (Table 4).

There was no difference with regards to pericardial effusions at baseline, 1 year, and 2 year between the two groups (*P* > 0.05). WHO FC was also not significantly different between two groups (*P* > 0.05) (Table 5).

## 4. Discussion

Our study is a single-center, open-label prospective controlled study, and we believe that it is the first time to study the effects of transition between bosentan and ambrisentan in PAH. Our data suggested that switching from a selective ETRA ambrisentan to an unselective ETRA bosentan may be reasonable and safe.

After transition, there was no marked change in hemodynamics as assessed by echocardiography, or in hematologic parameters, 6MWD, and WHO FC. Cost is commonly cited as a reason for transition to bosentan by Chinese patients. There could be potential bias on the outcomes of the patients because of the unevenness of the socioeconomic status of the patients and the discrepancy of the drug price. In fact, the socioeconomic factors have been balanced to be similar to each other amongst all the patients before their enrollment in this study. Even so, we did not ignore the role of economic factors, as the effect of drug prices on treatment exists in China. Also, if there is intolerance to ambrisentan, transition to bosentan may be an effective choice.

Although there was a statistical difference at baseline in 6MWD between the two groups (Table 4), hemodynamics (RHC) and other parameters (i.e., parameters of echocardiography, hematology, and WHO FC) were not statistically different. Because the grouping was carried out one year later, we did not guarantee that no difference existed in baseline patients. There was no difference between two groups after 1 year or 2 years; moreover, no significant change was seen in deviation from 1 year to 2 year between 2 groups. So we believe that the transition was successful.

Many studies have used 6MWD as the primary end point [7]. 6MWD remains the only endpoint tool for evaluation of treatment efficacy in PAH approved by FDA and European agencies. But it is well documented that 6MWD may be affected by subjective factors and may have a ceiling effect [8, 9]. Moreover, a previous small case series showed no change in 6MWD more than a year after transition [10]. Unlike other diseases, that is, chronic obstructive pulmonary disease and idiopathic pulmonary fibrosis, in PAH patients, there is no validated clinically important minimal difference or change in the 6MWD [11–13]. Due to these reasons, we measured the WHO FC as another effective functional index. FC is an important end point tool [14], as a supplement to the exercise capacity. In these patients, WHO FC was unchanged (*N* = 5/2/1 and 11/1/0, resp.). Thus, bosentan is similar to ambrisentan in maintaining stability of exercise capacity.

NT-proBNP and uric acid levels are biomarkers of heart failure, and their high plasma levels have consistently been reported as predictors of mortality [15–18]. Significant elevations of these biomarkers were not seen in the bosentan group or ambrisentan group.

The evaluation of pulmonary arterial hypertension requires a multimodality approach. Echocardiography continues to be a valuable tool to evaluate disease progression as it generates a wealth of information about response of the right heart to elevated pulmonary pressure. Numerous measurements can be used to identify alterations in right heart morphology, pressure, and function. Although each variable in isolation may have little utility, meaningful information is revealed when multiple parameters are considered together [19]. In this study, all the parameters, SPAP, TAPSE, LV eccentricity index, and pericardial effusion, were relatively unchanged with no statistical difference between the two groups. This implicated that the transition between the two drugs did not alter the hemodynamics. Transition from one ETRA to another may be required in different clinical situations. This may be due to side effects, liver function abnormalities, or availability of the drug. McGoon et al. have previously shown that patients who discontinued bosentan due to liver function test abnormalities tolerated ambrisentan and this change resulted in improvement in walk distance [20]. Our data were obtained from patients with normal liver function. We did not see abnormal liver function even after transition to bosentan. But the sample of this study is small. Regardless of this, the results of the study did not demonstrate any side effect including hepatic impairment on these 8 patients. Although the major adverse effect of bosentan is hepatic impairment [21], the severe hepatic impairment caused by bosentan is rarely reported in Chinese patients. Hence, the safety of bosentan is reliable. We do not recommend routine switch between ETRAs without compelling reasons such as side effects that must be mitigated. The only other reason for transition is perhaps the cost in low-income areas. Our study is only to provide an option for patients and clinicians as well that switch from ambrisentan to bosentan due to side effects or cost is safe and without hemodynamic deteriorations.

## Figures and Tables

**Table 1:** Demographics and baseline patient characteristics.

Patient number	Age (yr)	Gender	Race	BSA (m^2^)	PAH etiology	PAH medications	Duration of illness at transition (yr)	Group
1	46	Female	Chinese	1.63	CTD associated PAH	Sildenafil	1.0	Bosentan
2	64	Male	Chinese	1.79	CTD associated PAH	Sildenafil	0.7	Bosentan
3	44	Female	Chinese	1.50	HPAH	None	0	Bosentan
4	29	Male	Chinese	1.55	HPAH	None	0	Bosentan
5	64	Female	Chinese	1.70	CTD associated PAH	Sildenafil	2.3	Bosentan
6	58	Female	Chinese	1.55	IPAH	Sildenafil	2.2	Bosentan
7	45	Female	Chinese	1.55	CTD associated PAH	Sildenafil	0	Bosentan
8	59	Female	Chinese	1.50	IPAH	Sildenafil	0	Bosentan
9	54	Female	Chinese	1.44	IPAH	None	2.8	Ambrisentan
10	57	Female	Chinese	1.46	CTD associated PAH	None	0	Ambrisentan
11	30	Female	Chinese	1.53	CTD associated PAH	None	0	Ambrisentan
12	33	Female	Chinese	1.48	IPAH	Sildenafil	0.2	Ambrisentan
13	72	Male	Chinese	1.66	IPAH	None	0	Ambrisentan
14	18	Male	Chinese	1.59	CHD associated PAH, repaired	Sildenafil	0	Ambrisentan
15	18	Female	Chinese	1.45	CHD associated PAH, repaired	None	0	Ambrisentan
16	35	Female	Chinese	1.59	CHD associated PAH, repaired	None	0	Ambrisentan
17	33	Female	Chinese	1.53	CHD associated PAH, repaired	None	0	Ambrisentan
18	22	Female	Chinese	1.39	IPAH	None	0	Ambrisentan
19	40	Female	Chinese	1.46	IPAH	None	0	Ambrisentan
20	59	Female	Chinese	1.50	IPAH	Sildenafil	0	Ambrisentan

PAH, pulmonary arterial hypertension; IPAH, idiopathic pulmonary arterial hypertension; CTD, collagen tissue disease; CHD, congenital heart disease; BSA, body surface area.

**Table 2:** Baseline hemodynamic data of patients.

Patient number/group	mSVCP (mmHg)	mRAP (mmHg)	RVEDP (mmHg)	mPAP (mmHg)	CO (L/min)	CI (L/min/m^2^)	PVR (Wood unit)
Bosentan group	6.63 ± 3.02	6.63 ± 3.02	12.50 ± 4.38	59.13 ± 11.18	3.72 ± 0.73	2.34 ± 0.48	15.44 ± 4.70
Ambrisentan group	7.08 ± 3.92	7.08 ± 4.12	11.42 ± 4.74	62.58 ± 18.09	3.92 ± 0.92	2.38 ± 0.58	14.49 ± 7.03

CI, cardiac index; CO, cardiac output; mPAP, mean pulmonary artery pressure; PVR, pulmonary vascular resistance; mRAP, mean right atrial pressure; RVEDP, right ventricular end diastolic pressure; mSVCP, mean superior vena cava pressure; RHC, right heart catheterization; *P* > 0.05.

**Table 3:** Serum aminotransferase concentrations in all patients.

	Baseline		1 year		2 year	
	Bosentan	Ambrisentan	Bosentan	Ambrisentan	Bosentan	Ambrisentan
ALT (mU/mL)	24.9 ± 9.2	29.8 ± 14.4	23.3 ± 7.5	26.1 ± 8.0	26.6 ± 9.9	23.0 ± 9.1
AST (mU/mL)	26.3 ± 7.2	21.9 ± 4.9	26.3 ± 9.7	23.3 ± 7.3	26.9 ± 8.4	23.5 ± 6.1

*P* > 0.05.

**Table 4:** Baseline and follow-up data of parameters of echocardiography, hematology, and 6MWD.

	Baseline		1 year		2 year		Difference 1		Difference 2	
	Bosentan	Ambrisentan	Bosentan	Ambrisentan	Bosentan	Ambrisentan	Bosentan	Ambrisentan	Bosentan	Ambrisentan
6MWD (m)	432.5 ± 79.1	511.7 ± 41.4^∗^	422.3 ± 81.1	517.3 ± 60.8	409.0 ± 92.0	520.0 ± 72.4	10.3 ± 14.5	−5.6 ± 38.9	13.3 ± 29.6	−2.8 ± 21.2
NT-proBNP (pg/ml)	805.1 ± 440.4	516.6 ± 556.3	960.8 ± 673.8	604.7 ± 1247.6	1113.8 ± 902.3	334.3 ± 535.7	−155.4 ± 700.7	−104.8 ± 1375.0	−153.0 ± 376.9	270.3 ± 770.2
SPAP (mmHg)	76.0 ± 43.3	95.3 ± 31.8	71.5 ± 44.9	89.2 ± 38.7	87.8 ± 42.2	83.4 ± 33.9	4.5 ± 20.0	6.2 ± 26.5	−16.4 ± 18.1	5.8 ± 20.9
TAPSE (cm)	1.7 ± 0.4	1.8 ± 0.3	1.8 ± 0.4	1.8 ± 0.3	1.8 ± 0.4	1.8 ± 0.3	−0.14 ± 0.38	−0.01 ± 0.30	0.05 ± 0.21	0.06 ± 0.28
LV EI	1.33 ± 0.32	1.42 ± 0.33	1.28 ± 0.29	1.44 ± 0.36	1.34 ± 0.26	1.28 ± 0.25	0.05 ± 0.16	−0.02 ± 0.49	−0.06 ± 0.23	0.17 ± 0.20
D-dimer (ng/ml)	332.9 ± 204.7	275.4 ± 240.2	218.5 ± 96.0	255.8 ± 153.8	205.6 ± 108.0	301.8 ± 349.7	114.4 ± 135.3	19.7 ± 174.7	12.9 ± 110.9	−46 ± 324.4
PaCO_2_ (mmHg)	91.7 ± 3.6	94.0 ± 4.5	91.2 ± 3.3	94.6 ± 2.9	92.6 ± 3.1	92.5 ± 5.0	−0.43 ± 1.26	0.61 ± 5.58	1.33 ± 3.04	−2.14 ± 5.65
Uric acid (umol/l)	415.1 ± 93.3	427.9 ± 176.6	368.8 ± 67.6	387.2 ± 92.3	398.9 ± 86.4	415.9 ± 129.8	46.4 ± 54.3	40.8 ± 109.7	−30.1 ± 41.3	−28.8 ± 90.4

Difference 1, deviation of 1 year and baseline; difference 2, deviation of 1 year and 2 year; 6MWD, 6-minute walk distance. ^∗^*P* < 0.05.

**Table 5:** Baseline and follow-up data of pericardial effusion and WHO FC.

	Baseline		1 year		2 year	
	Bosentan	Ambrisentan	Bosentan	Ambrisentan	Bosentan	Ambrisentan
Pericardial effusion (*n*)	2	1	1	0	1	0
WHO FC (*N*, 2/3/4)	5/2/1	9/3/0	5/2/1	11/1/0	5/2/1	11/1/0

WHO FC, World Health Organization function class; *n*, the number of patients who have pericardial effusion; *N*, the number of patients of WHO FC II, III, and IV.
